# Hydroxy-Safflower Yellow A Alleviates Osteoporosis in Ovariectomized Rat Model by Inhibiting Carbonic Anhydrase 2 Activity

**DOI:** 10.3389/fphar.2021.734539

**Published:** 2021-11-05

**Authors:** Yang Wang, Xiaoyan Li, Feifei Deng, Ruofeng Yin

**Affiliations:** ^1^ Department of Orthopedic Surgery, China-Japan Union Hospital, Jilin University, Changchun, China; ^2^ Department of Hospital Infection Management, Hospital of Stomatology, Jilin University, Changchun, China

**Keywords:** HSYA, CA2, osteoporosis, osteoclast, hydroxy-safflower yellow a

## Abstract

**Background:** To investigate the therapeutic effect of Hydroxy-safflower yellow A (HSYA) on rat’s osteoporosis and explore its potential mechanism of action.

**Methods:** Bilateral ovariectomized female rats (OVX) were used to establish a postmenopausal rat model of osteoporosis. HSYA was given as an intervention, and estradiol was used as a positive control. The levels of serum alkaline phosphatase (ALP), calcium ion (Ca^2+^), and inorganic phosphorus (IP) were used to detect bone loss. Three months after modeling, the rats were sacrificed and the rat’s ovaries, kidneys, tibia, and femur were used to calculate the organ index. The bone marrow of the femur of the rats was stained with Giemsa staining. The femur strength of rats was measured by INSTRON. The degree of osteoporosis was detected by pathological staining after decalcification of bone tissue. Predicted the main targets of HSYA in combination with bioinformatics, and the proteins related to osteoclast differentiation were detected in combination with western blotting. The effect of HSYA on the differentiation of RAW264.7 cells into osteoclasts was observed.

**Results:** The Giemsa staining and serum test results showed that the operation was successful and affected bone metabolism. In the bone strength test, HSYA significantly increased the maximum threshold of femoral load in rats. Pathological examination showed that tibial cartilage, trabecular bone, and cortex significantly increased after treatment with HYSA. The number of osteoblasts increased while the number of osteoclasts decreased—elevated levels of type I and III collagen. Autodock was used for molecular docking of potential targets of HSYA. qPCR and western blot were used to show that the expression levels of CA2 and osteoclast differentiation-related proteins were significantly decreased after HSYA treatment. Cell level results showed that HSYA could inhibit the activity of osteoclasts and the ability of RAW264.7 cells to differentiate into osteoclasts.

**Conclusion:** HSYA can inhibit the differentiation and formation of osteoclasts by inhibiting the expression of CA2 and relieving osteoporosis symptoms in OVX rats.

## Introduction

Osteoporosis (OP) is a common disease in the elderly. It is highly prevalent in menopausal and postmenopausal women, with a much higher prevalence in women than men (De Martinis et al., 2020; Shetty et al., 2020). Not only does it predispose patients to fractures, but it can also bring about problems such as reduced immunity (Li et al., 2019). OP is divided into primary and secondary forms. Primary osteoporosis accounts for 90% of all osteoporosis and includes postmenopausal osteoporosis (type I osteoporosis) and senile osteoporosis (type II osteoporosis). Secondary osteoporosis can be secondary to other diseases or medically induced (De Martinis et al., 2020). The risk of OP is more likely to occur in the spine and femur. It is characterized by reduced bone mass, bone mineral density (BMD), and loss of microstructural integrity and bone strength, leading to increased bone fragility and consequently increased fracture risk. At the cellular level, due to much higher bone resorption by osteoclasts than bone formation by osteoblasts. This is due to an imbalance in bone reconstruction, with horizontal bone absorption much higher than bone formation (Akkawi and Zmerly, 2018). The main drugs currently available for treating primary osteoporosis are oestrogens, calcitonin bisphosphonates, and isoproterenol. However, they all have varying degrees of adverse effects (Black and Rosen, 2016). In contrast, natural products contain many compounds with precise and complex activity structures, relatively high safety, with similar advantages to synthetic compounds, as well as lower price. They are considered one of the sources of future drugs (Luo et al., 2019). Therefore, there is an urgent need to discover anti-osteoporosis medicines of natural origin that are safe and effective with relatively straightforward action. Hydroxy-safflower yellow A (HSYA) is a significant component of the Chinese Traditional Medicine *Carthamus tinctorius L*. It has been used as an injectable for many years in clinical practice, mainly for antiplatelet and anti-myocardial ischemia. Studies have shown that HSYA can promote the differentiation of zebrafish bone marrow mesenchymal stem cells into osteoblasts. Promote osteoblast viability and bone collagen expression, inhibit bone resorption from promoting bone formation, and prevent and cure glucocorticoid-induced osteoporosis (Liu et al., 2018). However, it is unclear whether HSYA can help improve osteoporosis in postmenopausal rats, and the effects on osteoclast differentiation and bone resorption need to be further investigated. Carbonic anhydrase 2 (CA 2) is an early feature of osteoclast differentiation (Lehenkari et al., 1998), and CA2 may play an important role in osteoporosis, osteoclast differentiation, and bone resorption and is associated with bone differentiation-related proteins. Its activity is essential for optimal bone resorption (Riihonen et al., 2007). CA2 promotes the production of protons, which drastically acidify the resorption gap (Lindskog, 1997). *In vivo*, mutations in the CA 2 gene inhibit bone resorption and lead to osteosclerosis. This suggests that CA 2 plays a vital role in osteoclast differentiation or bone resorption (Lehenkari et al., 1998; Sundquist et al., 1999).

In this study, HSYA was used as the primary intervention factor to simulate the symptoms of postmenopausal osteoporosis in ovariectomized (OVX) rats to learn its pharmacological effect on osteoporosis. In addition, RAW267.4 cells were combined to conduct cell-level experiments to further study the mechanism of CA2 and osteoclast differentiation.

## Materials and Methods

### Construction of a Rat Model of Osteoporosis

Seventy 4-month-old SD female rats (weighing 200–250 g) were purchased from Shanghai JieSiJie Laboratory Animal Co., LTD. The osteoporosis model was established by removing the bilateral ovaries of SD rats. The adult female rats were operated on sterilely under anesthesia (0.8% sodium pentobarbital, 0.35 ml/100 g, intraperitoneal injection), fixed in the supine position and the abdominal cavity was entered through a median abdominal incision. After 6 h of post-operative fasting, the animals were allowed to eat and drink freely. The animals were kept at room temperature in a well-ventilated, SPF-rated environment for 3 months. No surgery was performed on the control group. No ovaries were removed in the pseudo-operative group, and only the tissue was cut and sutured. The model and administration groups detected vaginal secretions during the estrous period after the operation and started the administration after the success of modeling was verified. When the experimental animals lost 15–20% of their body weight rapidly, could not feed themselves, had infected body organs, and did not respond well to drugs, they were euthanized by injection with excessive phenobarbital sodium.

The Experimental Animal Ethics Committee approved the animal experiment protocol of the China-Japan Union Hospital of Jilin University (No. SY20190506).

### Animal Grouping and Dosing Methods

One week after de-ovulation, vaginal exfoliative cell smears were performed daily, once per day, for 5 days, and the modeling was considered successful of the estrous cycle phase. The rats were randomly grouped into 10 rats/group, i.e., sham-operated group, model group, positive drug group (estradiol, E2, cas no. 53866-33-4), HSYA low-dose group (6 mg/kg, HSYA^Lo^), HSYA high-dose group (18 mg/kg, HSYA^Hi^), and the same batch of female rats as a normal control group. Estradiol and HSYA were purchased from Shanghai Maclin Biological Reagent Co., LTD. Both the sham (Sham) and the model groups were gavaged with saline 6 times a week, and the positive control group was subcutaneously injected with 30 μg mg/kg/d estradiol (Li et al., 2002; Canpolat et al., 2010). Both doses of HSYA were administered by gavage 6 times a week. The whole experiment lasted for 12 weeks.

The feeding conditions of rats used a12 h daily cycle, of 21–25°C, and 40–60% humidity.

### Giemsa Stain Testing

WE dropped 20 μl PBS on a clean slide. The vaginal secretions of female rats in each group were dipped clockwise with a clean cotton swab to make smears, and then 100 μl PBS was added to the dried smears. After 30 s, twice times the volume of PBS was added, using an ear washing ball to mix the solution with PBS buffer. After 5 min of dying, they were rinsed with tap water for 3 min. After allowing air to dry, slides were observed under a microscope (400×, DM500, Leica) and photos were captured. After modeling, we carried out 5 consecutive days of examination, and if they were not at the estrous cycle phase, this indicated a successful debridement procedure. At the end of the experiment, smears were made directly on each group of rat’s right tibial bone marrow cells and stained with Giemsa (cat no. G1015, Solarbio, China).

### Biochemical Testing Indicators

Blood was obtained through the posterior orbital plexus vein in rats, and peripheral blood was collected from each treatment group, centrifuged, and serum levels of ALP (cat no. BC2145), Ca^2+^ ions (cat no. BC0725), and inorganic phosphorus (cat no. BC1655) were determined. For details, refer to the instructions from Beijing Solarbio Co., Ltd. During the experimental period, tests were performed once a month to assess the degree of osteoporosis by the above biochemical indicators (Seif, 2014; Mukaiyama et al., 2015).

### Visceral Index and Bone Index

At the end of the experiment, the uterus, spleen, left kidney, right kidney, left tibia, left femur, right tibia, right femur, and the whole body of the rats were weighed. The index was calculated using the following formula: organ index = mean organ weight (mg)/mean body weight (g).

### Bone Strength Testing

The right femur of the rat was taken for bone strength testing. Each femur was placed between two fixation holes in an anvil of a universal test machine (INSTRON, Boston, US), and the compression was performed at a rate of 2 mm per minute drop. The computer detected the real-time pressure and the longitudinal coordinate of the point where the pressure value rose to a specific value and then fell unexpectedly. This was the pressure threshold at which the bone broke, i.e., the maximum load-bearing point of the right leg femur.

### Tissue Embedding

The left tibia and left femur of rats were fixed with neutral formaldehyde solution. After 24 h, we transferred them to neutral formaldehyde decalcification solution containing 25% EDTA-Na_2_ at 4°C for bone decalcification. We changed the decalcification solution daily until the done was completely decalcified. Judgment criteria: There was no block feeling until the 26G needle was inserted into the bone. After recalcitration, gradient dehydration was carried out with 75, 85, 95, and 100% alcohol, respectively. Xylene was transparent for 20min; paraffin was dipped in wax at 58°C for 3 h, then embedded with a Leica embedding machine (HistoCore Arcadia H), and solidified overnight at 4°C. The paraffin tissue was cut into tissue slices 5 μm thick using a Zeiss slicer (HM325, Germany) and baked overnight in a roasting machine at 55°C.

### HE Staining

The tissue slices were heated in an oven at 65°C for 20 min, then placed in xylene to melt wax for 20 min, and then subjected to 100, 95, 85, 75, and 50% ethanol for 2 min each time, and soaked in pure water for 1 min. They were then stained with hematoxylin solution for 3 min. After differentiation with 1% hydrochloric acid alcohol, 0.1% sodium bicarbonate was used to re-blue. We then used eosin for dyeing, dehydration using xylene for transparent, neutral gum for sealing. The results were observed under a light microscope (Leica, DM500).

### Osteoblast Staining

Osteoblast staining was performed using the alkaline phosphatase calcium-cobalt method. Bone tissue sections were dewaxed to water, incubation solution (prepare 2% calcium chloride 20 ml, 2% sodium barbiturate 10 ml, 3% β-sodium glycerophosphate 10ml, 5% magnesium sulfate 5 ml, distilled water 5 ml, incubate the solution, with the final pH of 9.4, and stored in the refrigerator) was added and incubated at 37°C for 30 min. It was then rinsed with running water for 2 min, incubated with 2% cobalt nitrate solution for 2 min, washed by immersion in distilled water, and finally incubated with 1% ammonium sulfide solution for 1 min, then washed with distilled water and sealed with glycerol gelatine. The enzyme active site shows a brownish-black color for cobalt sulfide precipitation, which is the osteoblast. All reagents used in the experiment were purchased from the Shanghai Maclin Reagent Co., LTD.

### TRAP Acid Phosphatase Staining

The bone tissue slices were dewaxed to water and incubated in TRAP incubation solution at 37°C for 50 min. The osteoclasts were observed to be wine red under the microscope. Ultra-pure water was used for quick washing before hematoxylin redyeing for 10 min, hydrochloric-ethanol color separation for 7 s, step by step dehydration, xylene transparent, and tablet sealing. Results were observed by microscope observation. The positive cells appeared wine red, which indicated osteoclasts.

For the preparation of TRAP incubation Solution A, 1.3608 g of Sodium acetate was added to 100 ml distilled water and dissolved with glacial acetic acid to adjust the pH value to 5.0. Solution B used hexazo accessory fuchsin, 4% sodium nitrite, 2 g sodium nitrite, and 50 ml deionized water. Vice fuchsin solution used 2.5 g Vice fuchsin, 50 ml deionized water, and 7.5 ml of concentrated hydrochloric acid heated to 90°C, dissolved, filtered, and stored at 4°C in a brown bottle. The first 4% sodium nitrite was mixed with accessory fuchsin solution in equal proportion. Solution C used 20 mg Naphthol AS-BI phosphate dissolved with 1 ml N, N-dimethylformamide. For the working solution, we mixed 18 ml of solution A, 1 ml of solution B, and 1 ml of solution C, and adjusted the pH to 5.0 with 1 M NaOH or 1 M hydrochloric acid, then added 0.282 g of sodium potassium tartrate and allowed it to fully dissolve. It was then filtered and then use as the TRAP incubation solution. All reagents used in the experiment were purchased from Shanghai Maclin Reagent Co., LTD. 

### Sirius Red Staining

For Collagen fiber determination, sections were dewaxed in water, stained in Sirius red solution for 1 h, rinsed in tap water for 1 h, then nucleated by hematoxylin, routinely dehydrated, sealed with clear gum, and observed with an Olympus polarised light microscope (DM500, Leica, with polarizer installed on the aperture of the microscope). The nuclei were then stained with hematoxylin for 1 h, dehydrated, and sealed with clear gum. Two types of collagen fibers could be seen under the polarised light microscope. Type I collagen fibers were closely packed and showed strong birefringence, with yellow or red fibers. Type III collagen showed weak birefringence, with fine green fibers. It carried out the specific procedures of this experiment according to the instructions of Shanghai MKbio Biological Technology Co., LTD. Sirius red Staining Kit (cat no. MM1004). The experimental results were measured by Image-Pro Plus 6.0 (IPP 6.0), and the results were presented in the form of area proportion.

### Molecular Simulation Docking

The 3D structure of HSYA was downloaded from the ChemSpider database (http://www.chemspider.com/). The format was adapted to PDB using Pymol software. The 3D form of the CA2 protein was downloaded from the RCSB Protein Data Bank (http://www.rcsb.org/) (PDB ID: 3ML2). The water molecules were removed separately from the CA2 protein using Pymol software, and the ligand sites were extracted and then saved the separately from the CA2 protein. The position parameters of the ligand were obtained after converting all formats to PDBQT format using Autodock. Autodock vina 1.1.2 was used for molecular docking studies. Based on the position of the protein-ligand, the coordinates of the protein active site were determined, and default values were used for all other parameters. Finally, the conformation with the lowest docking binding energy was selected for docking binding energy pattern analysis and then visualized using Pymol molecular simulation software.

### Western Blot

Freshly obtained rat right femur tissue was placed in a mortar and pestle, ground to a fine powder with liquid nitrogen, and the tissue was lysed with RIPA lysis buffer (Sigma-Aldrich, MO, USA) containing protease and phosphatase inhibitors to extract the proteins. Protein concentrations were quantified using the Pierce™ BCA Protein Assay Kit (ThermoFisher, MA, USA), and proteins were then separated on a 10% SDS-polyacrylamide gel and transferred to PVDF membranes. Blotted membranes were closed with TBST containing 5% skimmed milk for 1 h. We then added rabbit anti rat antibody CA2 (cat no. ET1706-47, Huabio, China), OPG (cat no. R1608-4, Huabio), RANK (cat no. ER 1915-70, Huabio), RANKL (cat no. A2550, ABclonal, China), MMP-9 (cat no. A2095, ABclonal), CTSK (cat no. A1782, ABclonal), TRAF6 (cat no. A0973, ABclonal), NFATc1 (cat no. A1539, ABclonal), and c-fos (cat no. A0236, ABclonal). This was followed by incubation with goat anti-rabbit IgG H&L (HRP) secondary antibody (cat no. AS014, ABclonal, China). Rabbit anti rat antibody GAPDH (cat no. A19056, ABclonal) was used as the internal reference for normalization. The UVP immunoblot detection system (ChemiDoc-It Imaging System, Upland, USA) detected the protein bands and quantified them on a greyscale by Image J software.

### Quantitative Real-Time PCR

Total RNA was extracted from liquid nitrogen milled right leg femur tissue (Invitrogen, Carlsbad, CA, USA) using TRIZOL reagent. Reverse transcription cDNA synthesis kits (ThermoFisher, Rockford, USA) were used. Real-time fluorescent quantitative PCR was performed using an SYBR Green PCR kit (ThermoFisher, Rockford, USA) under a 7,500 Fast system (ABI, CA, USA). The qPCR reaction conditions were pre-denaturation at 95°C for 5 min, denaturation at 95°C for 10 s, annealing at 60°C for 30 s, a total of 40 cycles, followed by fluorescence signal detection at annealing. GAPDH was used as an internal reference for normalization.

The primer sequence was as follows:

CA2: Forward- 5′- TCC CTA CAG CCT CTG CTC ATA-3′, Reverse- 5′-ATG AGC CCC AGT GAA AGT GAA A-3`.

OPG: Forward-5′-GTG CAC TCC TGG TGT TCT TG-3`, Reverse- 5′- TGC CAG GAG CAC ATT TGT CA-3`.

RANKL: Forward- 5′- CAC AGC GCT TCT CAG GAG TT-3`, Reverse- 5` - ATG GTG AGG TGA GCA AAC GG-3`.

RANK: Forward- 5′- CAT AAA GTC TGT GAT GCA GGC AAG-3′, Reverse-5′-ACA CGG TATC CAC GTT GAG CTG-3`.

GAPDH: Forward- 5` -ATC ACT GCC ACC CAG AAG-3′, Reverse- 5` -TCC ACG ACG GAC ACA TTG-3`.

### Osteoclast Induction

For specific methods, refer to the study of Li et al. (2013). RAW264.7 cells (1 × 10 3 cells/well, purchased from Zhejiang Ruyao Biotechnology Co., LTD.) were laid in 96-well plates and treated with 100 ng/ml RANKL for osteoclast induction.

### MTT Detects Cell Activity

RAW264.7 cells (1 × 10^3^ cells/well) were laid in 96-well plates and treated with 0, 0.25, 0.5, 1, 2 μM of HSYA and 10,50,100,200,500,1000 ng of mouse CA2 recombinant protein (Novoprotein co., LTD., China) were cultured at 37°C for 48 h. After 48 h, 10 μl MTT (5 mg/ml, Beyotime biotechnology co., LTD., China) was added to each well and the plates were incubated at 37°C for 4 h. The medium was removed and 150 μl dimethyl sulfoxide (DMSO, Sigma) was added to each well. The plates were shaken for 5 min. Then a microplate reader (CMax Plus. Molecular Devices, USA) at 570 nm was used for absorbance measurements. Each concentration is provided with 3 complex wells.

### Osteoblast Staining

RAW264.7 cells (2 × 106 cells/well) were inoculated into 6-well plates, and cover glass slides were placed at the bottom of each well plate for cell slippage. After cell adhesion, 100 ng/ml RANKL was given for osteoclast induction, and 1 μM HSYA and 100 ng CA2 were given for treatment. The cell slides were removed 4 days later. The cells were fixed in 4% paraformaldehyde for 10 min for TRAP staining. For details, see TRAP staining for tissue parts. Photographs were taken using a light microscope (DM500, Leica).

### Exploration of Osteoclast Differentiation Mechanism

Western blotting was performed on cells treated with 1 μM HSYA and 100 ng CA2 for RIPA lysis. The specific procedure could be referred to as the operation in animal experiments. The target proteins were CA2, RANK, MMP-9, CTSK, TRAF6, NFATc1, and c-fos. GAPDH was used as the reference gene and normalized.

### Statistics Analysis

SPSS 22.0 (SPSS Software Inc., La Jolla) was used for statistical analysis. All data were expressed as mean ± SD, and the statistical differences among different groups were assessed by one-way analysis of variance. The two groups were compared using *t*-test. A *p* < 0.05 indicated a significant difference, and *p* < 0.01 indicated that there was a very significant difference.

## Results

### Effect of HSYA on the Physiological Parameters of Desiccated Rats

**FIGURE 1:**
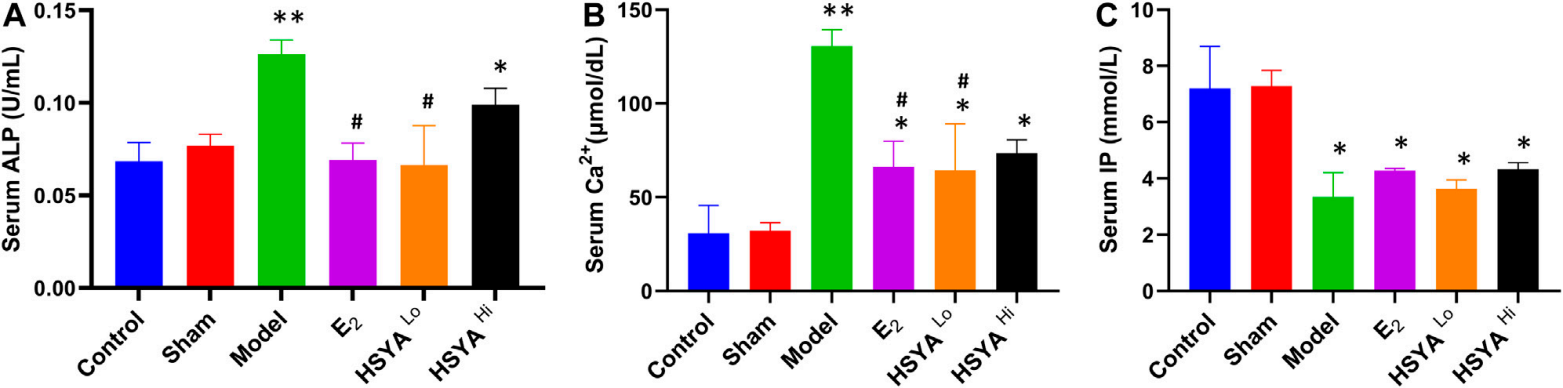
The results of serum **(A)** ALP, **(B)** Ca2+, and **(C)** IP biochemical tests. “**” means *p* < 0.01, “*” means *p* < 0.05, the difference is statistically significant compared with the control group. “^#^” means *p* < 0.05, compared with the model group, the difference is statistically significant.

The vaginal secretions of the rats were also stained with Giemsa stain for 5 days after surgery. The results showed (Supplementary Figure S1) that the epithelial cells of the vaginal secretions of the rats were reduced after OVX. Many leukocytes were present during the five consecutive days, with no kinetic phase occurring. To investigate the effect of HSYA on bone quality when given after OVX, we tested the serum of each group of rats for Osteoporosis-related biochemical parameters. The results showed (Figure 1A) that ALP levels were significantly higher in the model rats than in the control group (*p* < 0.01). After treatment with low dose HSYA, the increase in ALP levels significantly reversed, and the difference was statistically significant compared with the model group (*p* < 0.05). Compared with the control group, the difference between the two groups was not statistically significant. ALP levels in the high-dose HSYA group decreased. The difference was not statistically significant compared with the model group but was statistically significant compared with the normal control group (*p* < 0.05). Serum concentrations of Ca ions and inorganic phosphorus (IP) were also measured to investigate the anti-bone resorption effects of HSYA. Elevated serum calcium levels indicated increased bone resorption (Figure 1B). However, IP levels in the model group were significantly lower (*p* < 0.01) compared to the control group (Figure 1C). The IP content of the drug-treated group was not significantly different from that of the model group. Ca ion levels were significantly reduced after treatment with low doses of HSYA compared to the model rats, and the difference was statistically significant (*p* < 0.05). However, there was no significant difference between the treatment groups with high HSYA and low doses (*p* > 0.05).

### Effect of HYSA on Organ Index in Osteoporotic Rats

**FIGURE 2:**
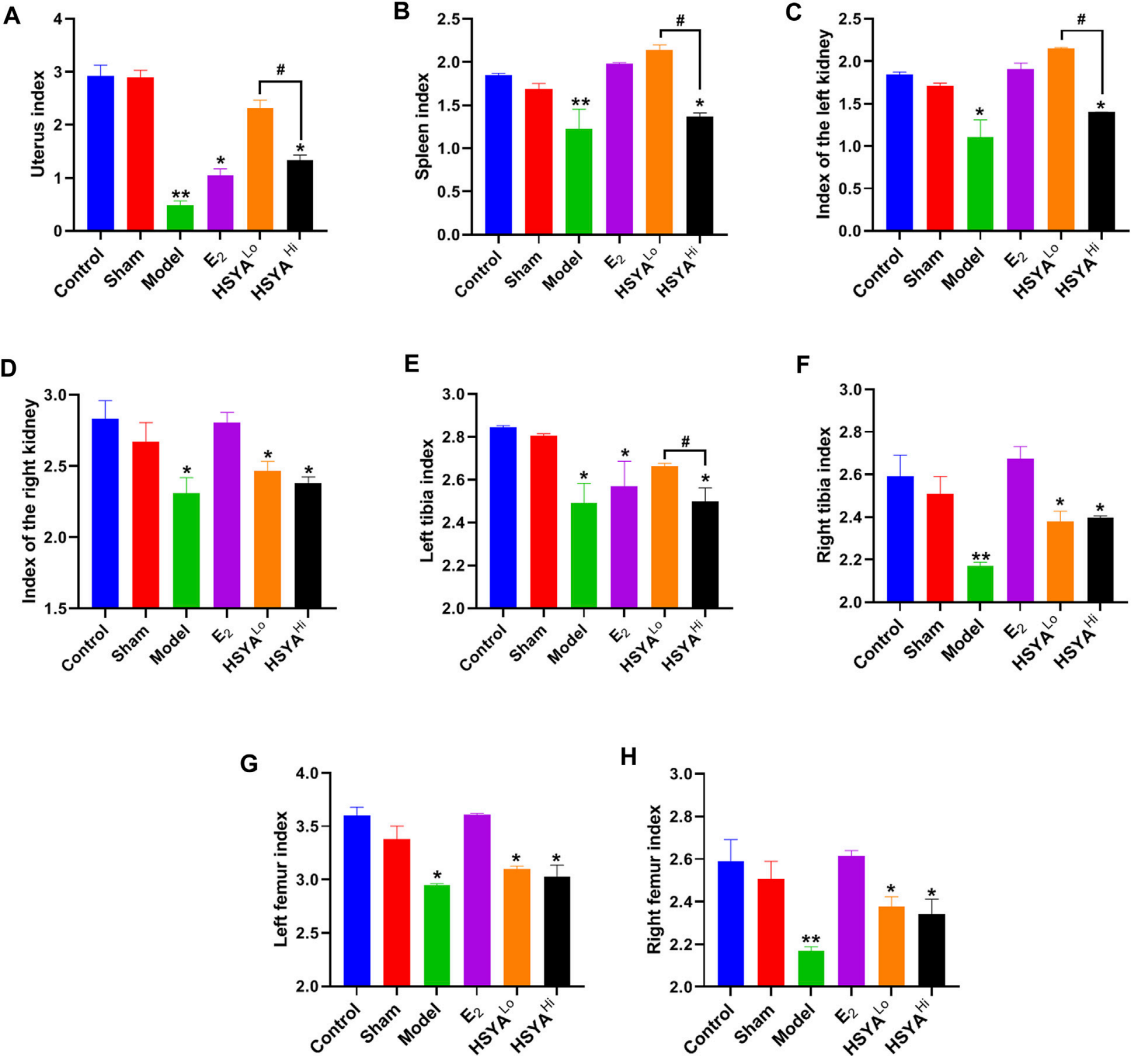
Index of various organs in rats. **(A)** rat uterine index. **(B)** rat spleen index. **(C)** rat left kidney index. **(D)** rat right kidney index. **(E)** rat left leg tibia index. **(F)** rat right leg tibia index. **(G)** rat left leg femur index. **(H)** rat right leg femur index. “**” indicates *p* < 0.01, “*” indicates *p* < 0.05, compared with the control group, the difference is statistically significant. “^#^” indicates *p* < 0.05, and the difference was statistically significant when compared with the HSYA group.

All rats were sacrificed after the experiment uterus of each group was observed (Supplementary Figure S2). It was evident that the uterus of the rats showed significant atrophy after OVX and that the uterus of normal rats was whole and elastic. The uterine index was calculated and was significantly lower (*p* < 0.05) in the OVX model group (uterine wet weight/body weight) compared to the control group (Figure 2A). The splenic index, renal index, tibia index, and femur Index were significantly lower in the model group than in the control group (*p* < 0.05). The administration of estradiol was able to alleviate this downward trend. As can be seen from Figures 2A–C, low concentrations of HSYA significantly improved the uterine index, spleen index, and left kidney index (*p* < 0.05), and the effect was significantly higher than that of high concentrations of HSYA (*p* < 0.05). At the same time, there was no significant difference in the right kidney index (*p* > 0.05) Figure 2D. In addition, the left tibia index (Figure 2E) was significantly higher in the low concentration HSYA treatment group than in the high concentration and model groups. The difference was statistically significant (*p* < 0.05), while the femur index and right tibia index (Figures 2F–H) were not significantly different (*p* > 0.05).

### Effect of HYSA on Bone Strength in Osteoporotic Rats

**FIGURE 3:**
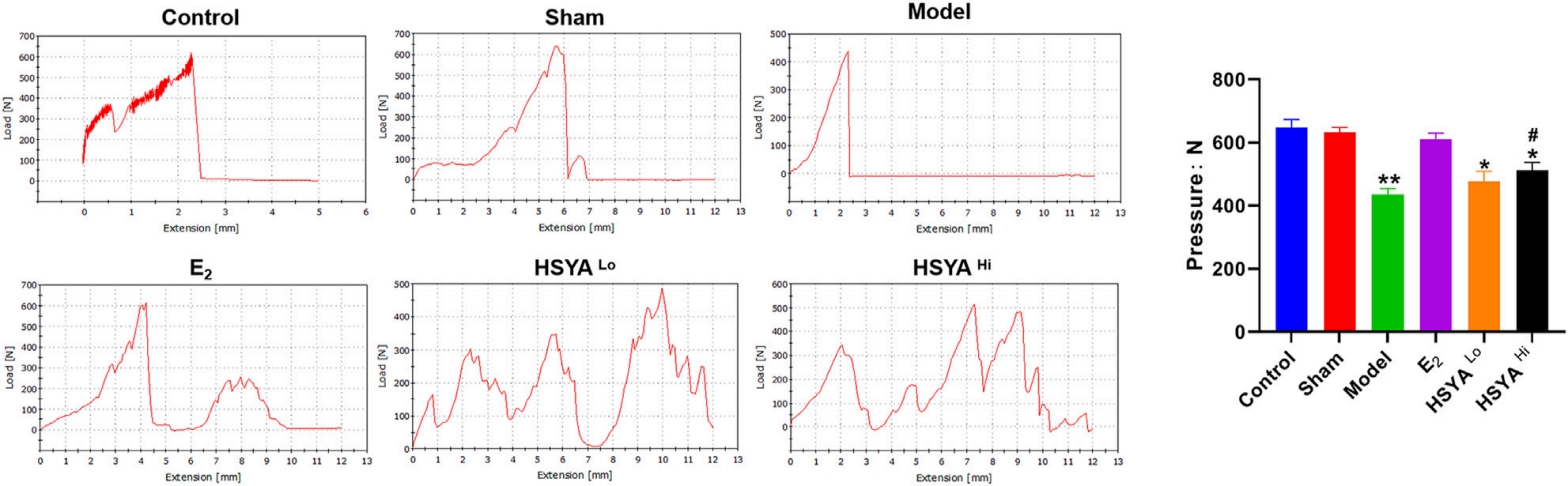
Maximum weight-bearing threshold of the right leg femur in rats tested by INSTRON, with quantified results on the right. The horizontal coordinates indicate the distance that the upper platen of the instrument continued to move downward after touching the femur. “**” indicates *p* < 0.01, “*” indicates *p* < 0.05, and the difference is statistically significant compared to the control group. “^#^” indicates *p* < 0.05, and the difference is statistically significant when compared with the model group.

The right leg femur of each group of rats was tested for weight-bearing using the INSTRON Apparatus (see Supplementary Figure S3 for instrumentation and operation) to assess the effect of HSYA on the Ultimate load pressure bone (Figure 3). The results showed that compared to the maximum load-bearing value of 648 ± 25N in the control group, the final load-bearing threshold of the right leg femur in the model group was significantly reduced to 436 ± 19N, with a statistically significant difference between the two groups (*p* < 0.05). Compared to the model group, the weight-bearing threshold of the right femur of the rats was increased to 512 ± 25N and 478 ± 31N. After 6.18 mg/kg HSYA treatment, with a significant difference between the 18 mg/kg HSYA treatment group and the model group (*p* < 0.05).

### Effects of HYSA on Bone Morphology and the Pathology in Osteoporotic Rats

**FIGURE 4:**
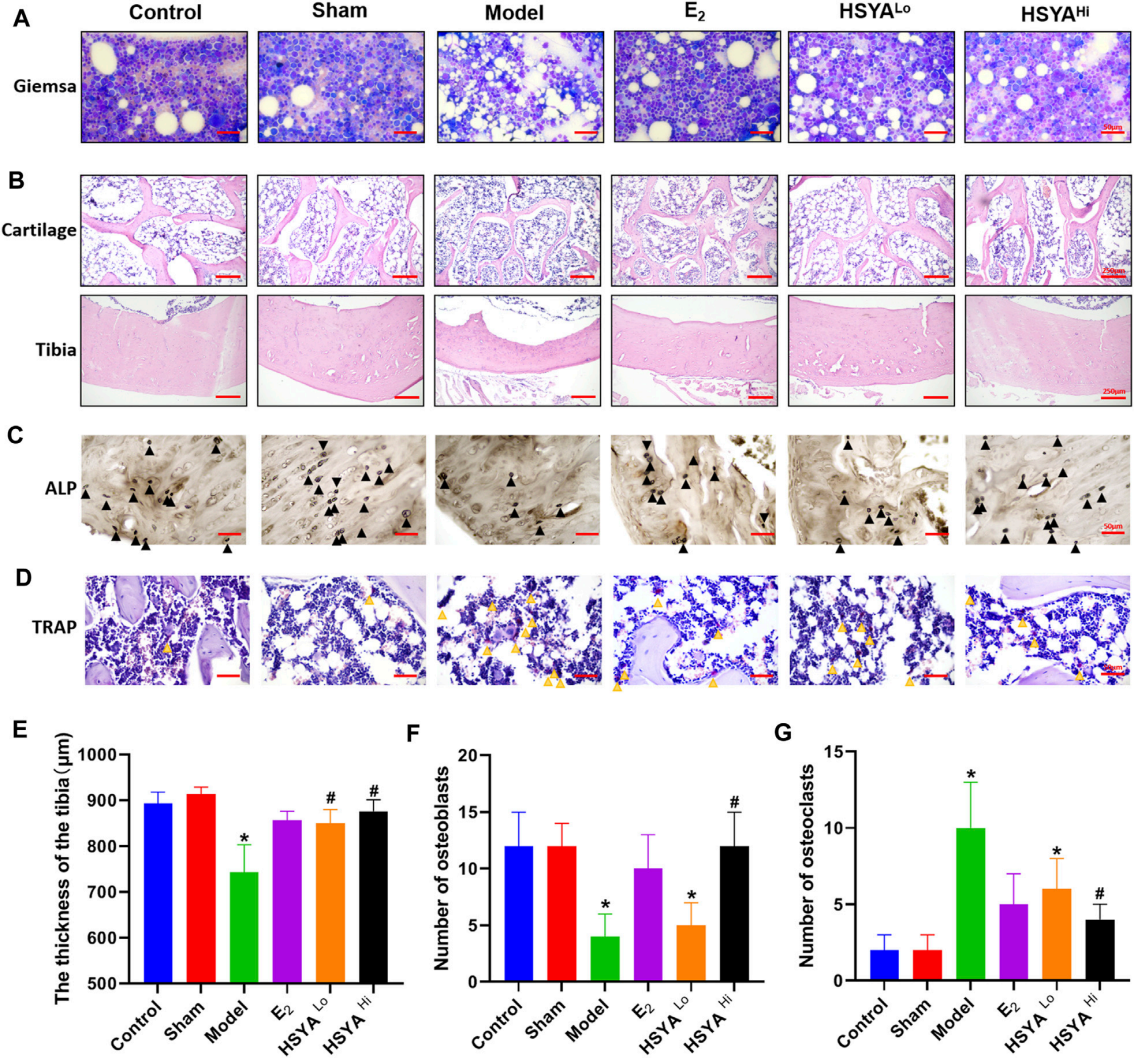
Protection of bone quality by HSYA in osteoporotic rats. **(A)** Giemsa staining of bone marrow cells in the left leg tibia of rats, the vacuoles in the figure are adipose tissue. **(B)** HE staining of the proximal femoral cartilage of rats in the upper panel, and HE staining of the tibia of rats in the lower panel. **(C)** alkaline phosphatase staining of osteoblasts, the black triangles in the figure are osteoclasts. **(D)** TRAP staining for osteoclasts, the yellow triangle in the figure is marked with osteoclasts, and the cells are burgundy. **(E)** the results of tibial bone thickness measurement in the left leg of rats. **(F)** the statistical graph of osteoblasts number. **(G)** the statistical graph of osteoclast number. “*” indicates *p* < 0.05, compared with the control group, the difference is statistically The difference is statistically significant compared with the control group. “^#^” indicates *p* < 0.05, the difference is statistically significant in HSYA treatment group compared with the model group.

**FIGURE 5:**
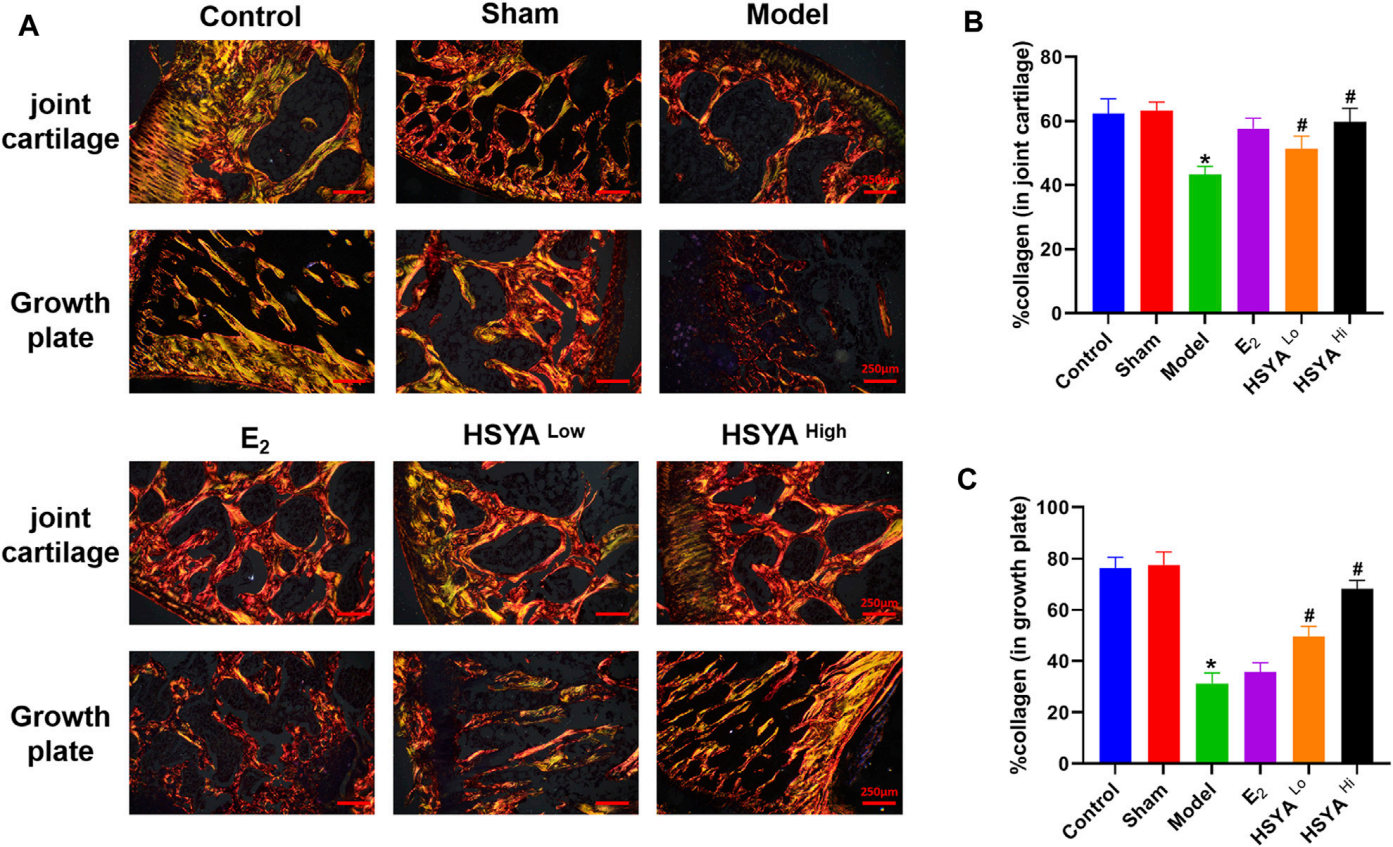
Sirius red staining of collagen. **(A)** After the epiphysis and growth plate were stained with Sirius red, the collagen was taken on the positive film. **(B)** IPP6.0 software quantified the fluorescence area of the epiphysis in red and yellow-green, and the results were quantified by the ratio to the total bone tissue area in the image. **(C)** IPP6.0 software quantified the fluorescent area of red and yellow-green at the growth plate, and the results were quantified by the ratio with the total area of bone tissue in the image.“*” indicates *p* < 0.05, compared with the control group, the difference is statistically The difference is statistically significant compared with the control group. “^#^” indicates *p* < 0.05, the difference is statistically significant in the HSYA treatment group compared with the model group.

The results of Giemsa staining showed an increase in fatty granules, a decrease in naive and primitive monocytes, and an increase in the content of mature monocytes in the bone marrow fluid of the model group (Figure 4A). The results of HE staining (Figure 4B) showed that the thickness of the tibia in the model group was 743 ± 60 μm, compared to 892.6 ± 25 μm in the control group, with a statistically significant difference (*p* < 0.05). The thickness of the tibia in the HSYA treatment group was 849 ± 31 μm and 876 ± 25 μm respectively, which were both significantly increased compared to the model group, with statistically significant differences (*p* < 0.05) (Figure 4E). The cartilaginous parts of the femur showed significantly thinner trabeculae in the model group. There were thicker and more numerous trabeculae in the control group. After a high dose of HSYA, the number of trabeculae in the bone tissue recovered, and the bone cortex thickened. The results of the alkaline phosphatase staining of osteoblasts (Figure 4C) showed that the high concentration of HSYA was able to significantly increase the number of osteoblasts (12 ± 3) compared to the number of osteoblasts (4 ± 2) in the model group, and the difference was statistically significant (*p* < 0.05) (Figure 4F). Tartaric acid staining of osteoclasts (Figure 4D) significantly increased the number of osteoclasts in the bone tissue of the model group to 10 ± 3 compared to 2 ± 1 osteoclasts in the control group with a statistically significant difference (*p* < 0.05) (Figure 4G). After treatment with 6, 18 mg/kg HSYA, osteoclasts were reduced to 6 ± 2 and 4 ± 1 under ×400 magnification, respectively. After Sirius red staining (Figure 5), polarised light microscopy photograph showed an increased appearance of type I and III collagen in the HSYA treated group compared to the lower type I and III collagen in the model group collagen sites appearing as red-yellow birefringence in the graph.

### HSYA Regulates the Expression of Osteoclastic Differentiation-Related Proteins

**FIGURE 6:**
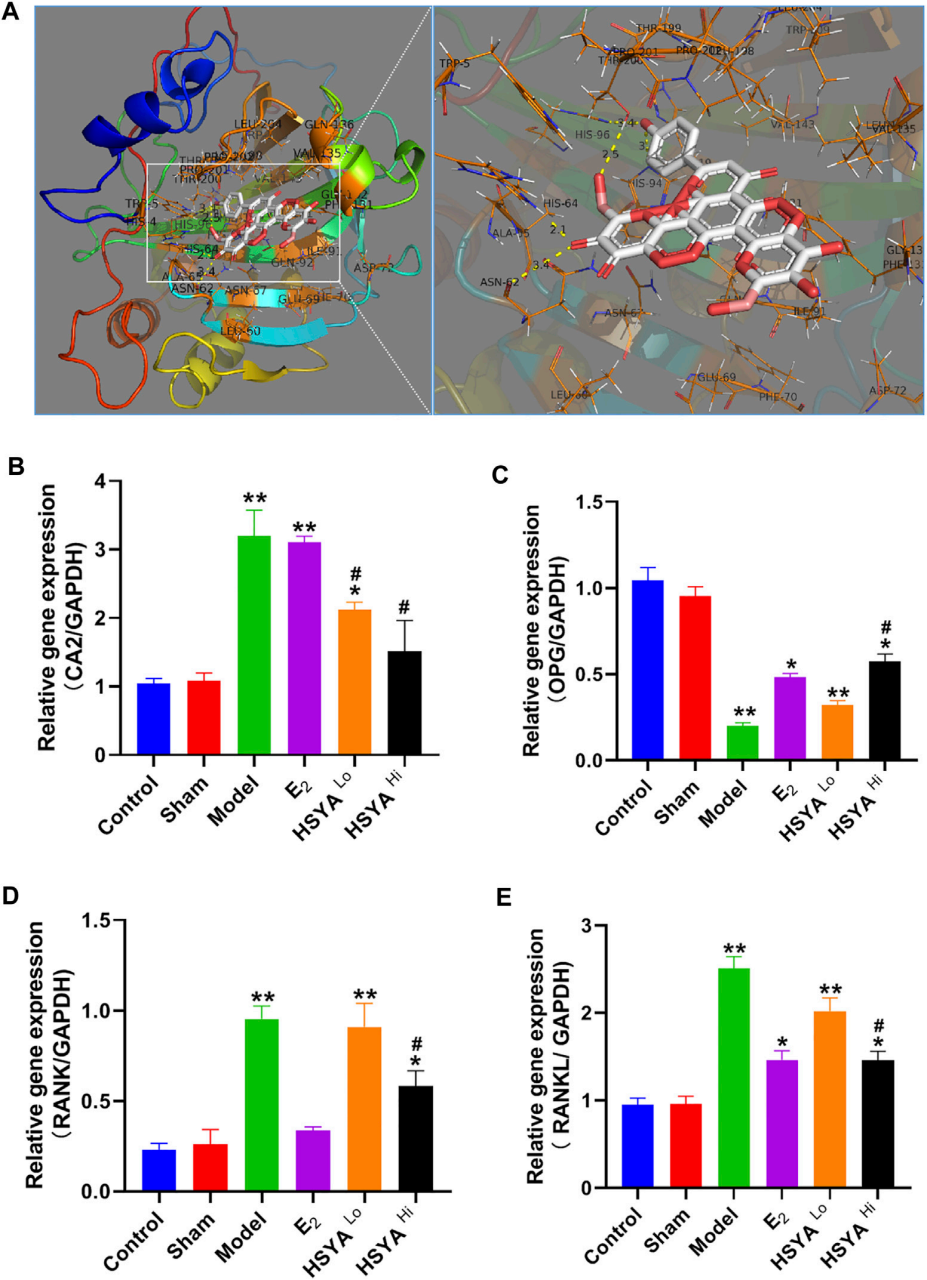
regulation of CA2 and osteoclast differentiation-related genes by HSYA. **(A)** Pymol molecular simulation of HSYA and CA2 docking results. **(B)** qPCR to detect the relative expression level of CA2 gene in rat femur. **(C)** qPCR to detect the relative expression level of OPG gene in rat femur. **(D)** qPCR to detect the relative expression level of RANK gene in rat femur **(E)** qPCR detected the relative expression level of RANKL gene in rat femur. “**” means *p* < 0.01, “*” means *p* < 0.05, compared with the control group, the difference is statistically significant. “^#^” indicates *p* < 0.05, the difference was statistically significant in the HSYA treatment group compared with the model group.

**FIGURE 7:**
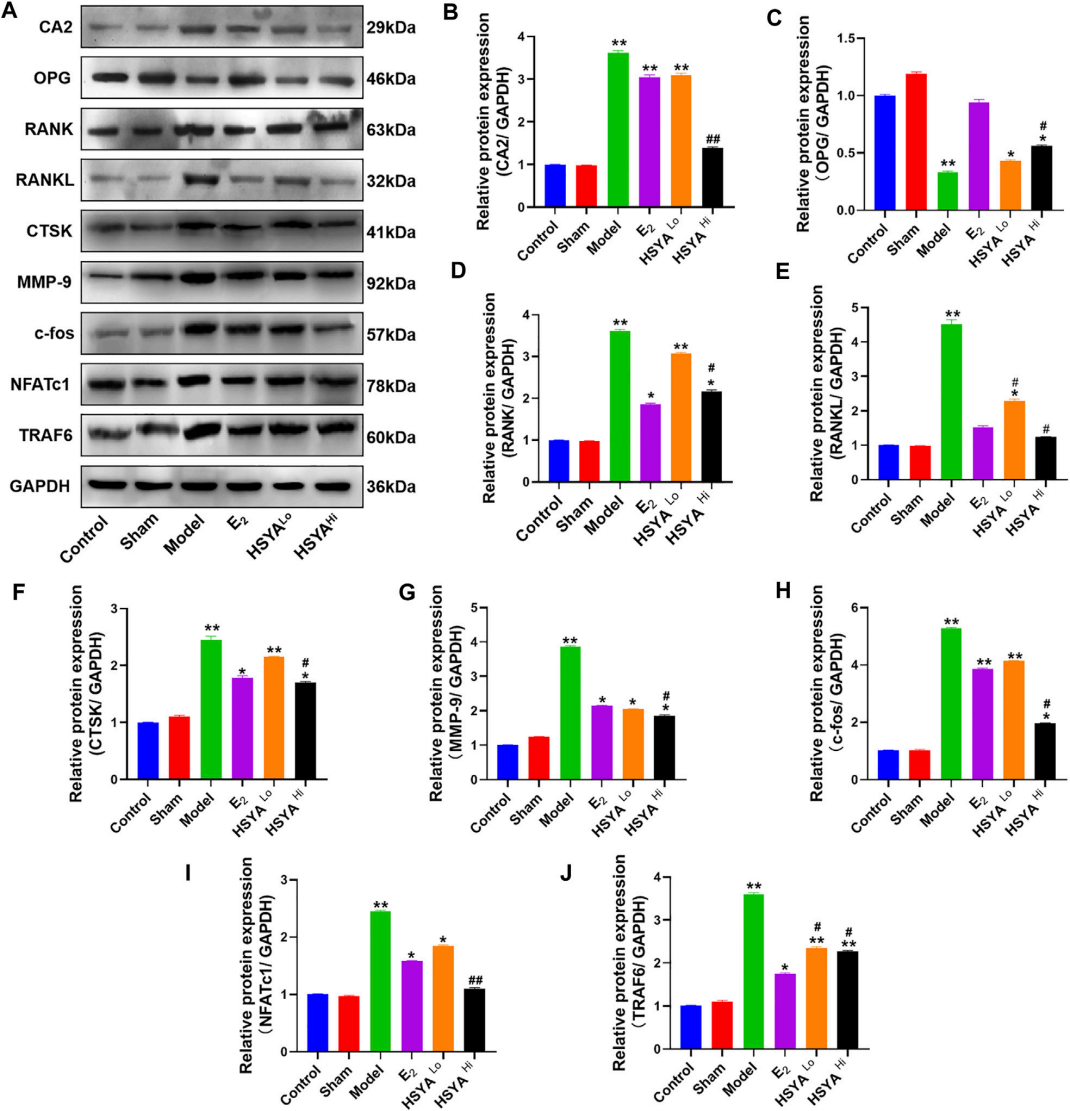
Regulation of CA2 and osteoblast differentiation-related proteins by HSYA. **(A)** The relative expression levels of CA2, OPG, RANK, RANKL, CTSK, MMP-9, c-fos, NFATc1, and TRAF6 proteins were detected by western blotting. **(B–J)** The gray values of CA2, OPG, RANK, RANKL, CTSK, MMP-9, C-FOS, NFATc1, and TRAF6 proteins were quantified by ImageJ software. “**” indicates *p* < 0.01, “*” indicates *p* < 0.05, and the difference was statistically significant when compared with the control group. Statistical significance. “^#^” indicates *p* < 0.05, and the difference was statistically significant in the HSYA treatment group compared with the model group.

The docking of HSYA to CA2 protein was simulated by Pymol software and found that HSYA had amino acid residue binding sites to the ligand sites of known inhibitors of CA2, with a binding energy of −9.5 kcal/mol (Figure 6A). qPCR results showed (Figure 6B) that the relative expression levels of the CA2 gene in the model group were significantly increased compared to the control group (*p* < 0.01). E2 administration had no significant effect on the relative gene expression level of CA2, and the difference was not statistically significant (*p* > 0.05). After treatment with different doses of HSYA, the gene expression levels of CA2 decreased significantly compared to the model group, and the difference was statistically significant (*p* < 0.05). The expression levels of OPG, RANKL, and RANK genes in bone tissue were also analyzed by qPCR (Figures 6C–E). The results indicated that the expression levels of OPG genes were significantly lower in the model group than in the control group (*p* < 0.05). The expression levels of RANKL and RANK genes were related to osteoclast differentiation, were significantly up-regulated. The differences were statistically significant (*p* < 0.05). Western Blot results (Figure 7) showed that compared with the control group, the left femur of model rats was significantly reduced in OPG protein, and the levels of CA2, osteoclast differentiation proteins RANKL, RANK, TRAF6, NFATc1, and osteolysis related proteins CTSK and MMP-9 were significantly increased (*p* < 0.05). HSYA reversed the decrease of OPG protein after OVX, and down-regulated the levels of CA2, osteoclast differentiation protein, and osteolysis related protein, with statistically significant differences, compared with the model group (*p* < 0.05).

### HSYA Regulates Osteoclast Differentiation Through CA2

**FIGURE 8:**
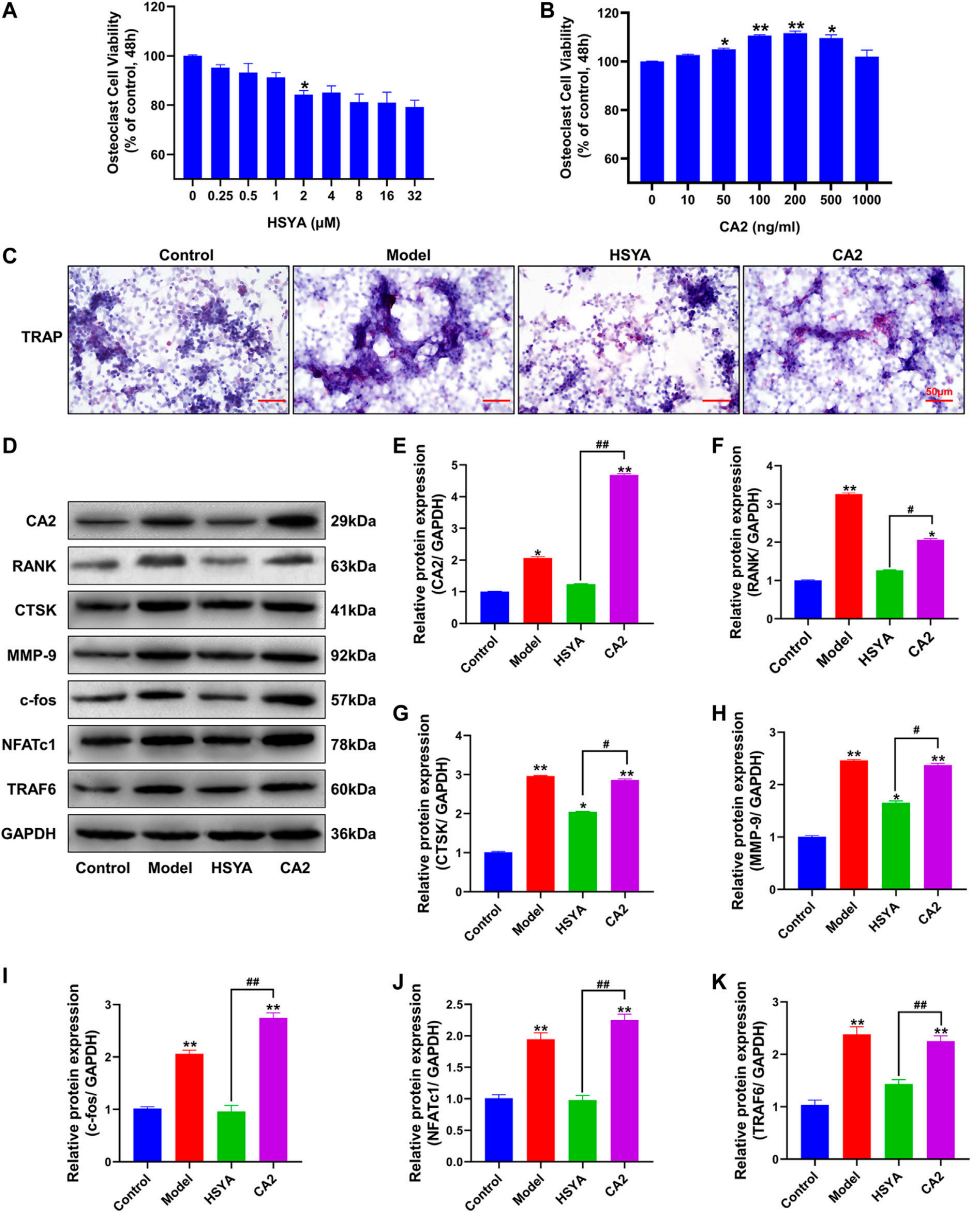
HSYA regulates osteoclast differentiation of RAW264.7 cells through CA2. **(A)** An MTT was used to detect the effects of different doses of HSYA on RAW264.7 cells. **(B)** MTT was used to detect the effects of Different concentrations of CA2 recombinant protein on RAW264.7 cells. **(C)** TRAP staining results of RAW264.7 cells. **(D)** The expression levels of CA2, RANK, CTSK, MMP-9, c-fos, NFATc1, and TRAF6 proteins were detected by western blotting. **(E–K)** The gray values of CA2, RANK, CTSK, MMP-9, c-fos, NFATc1, and TRAF6 proteins were quantified by ImageJ software. Compared to the control group, “**” indicates *p* < 0.01, “*” indicates *p* < 0.05. The HSYA group was compared with the CA2 group, “^##^” indicates *p* < 0.01, “^#^” indicates *p* < 0.05.

Figure 8A shows the effect of different doses of HSYA on the osteoclast activity measured by MTT. The results showed that osteoclast activity decreased significantly after 1 μM HSYA compared with 0 μM HSYA, and the difference between the two groups was statistically significant (*p* < 0.05). Figure 8B shows the effect of MTT detection of different doses of mouse recombinant protein CA2 on osteoclast activity. The results showed that 100 ng/ml of CA2 significantly promoted the activity of osteoclasts. Compared with 0 ng/ml of CA2, the difference between the two groups was statistically significant (*p* < 0.05). Therefore, the working concentrations of HSYA and CA2 used in subsequent experiments were 1 μM and 100 ng/ml, respectively. Figure 8C shows the results of the TRAP staining of osteoclasts. According to the results, the positive level of TRAP staining of osteoclasts in the model group was higher than that in the control group and HSYA group. After the intervention of CA2 protein, the inhibition of HSYA on TRAP positive osteoclasts could be effectively reversed. Combined with western blotting experiment (Figure 8D), the results showed that (Figures 8E–K, the statistical quantification results of western blotting gray value) HSYA could significantly inhibit the expression levels of osteoclast differentiation related proteins RANK, TRAF6, NFATc1, c-Fos, and bone degradation related proteins CTSK and MMP-9. Compared with the model group, the difference was statistically significant (*p* < 0.05). The inhibition of HSYA on osteoclast differentiation related proteins and bone degradation related proteins was reversed after 100 ng/ml CA2 intervention, and the difference between the two groups was statistically significant (*p* < 0.05).

## Discussion

Due to the presence of high bone transformation and consequent bone loss in OVX rats, as is the case in postmenopausal humans, it can be prevented by estrogen replacement. While human and rat skeletal responses to estrogen deficiency are strikingly similar, OVX rats are considered the gold standard model for evaluating drugs to prevent and reverse osteoporosis (Kharode et al., 2008). In this study, an osteoporosis model was established by removing the ovaries of female rats bilaterally. Firstly, we did this by examining the kinetic phase of the rats after de-ovulation, combined with Giemsa for staining the vaginal secretion smear. The results showed that within a week, the normal cells in the vaginal secretion were significantly reduced. The vast majority were leukocytes, indicating a dramatic decrease in the estrogen level of postmenopausal females. The anatomical experiments on the de-ovulated ovaries of all groups of rats showed evident atrophy, indicating that the OVX model was successfully established.

The serum biochemical results were mainly evaluated in the osteoporosis model by three indicators, Ca^2+^, P, and ALP content. This experiment showed that the blood Ca^2+^ level In the model group was higher than that in the control group, indicating a continuous loss of calcium ions from bone tissue and a decrease in bone quality. ALP is a ubiquitous intracellular enzyme that is present in osteoblasts and promotes bone mineral deposition by breaking down inorganic phosphate (Qi, 2018). In Stephen’s study (Coburn et al., 1998), inorganic phosphates act as competitive alkaline phosphatase inhibitors to inhibit serum ALP activity. Moreover, hypophosphatemia and elevated serum ALP levels were likely to lead to decreased bone mineral content and trabecular bone number (Zhang et al., 2021). The results showed that the level of ALP in the model group was higher than that in the control group, and the content of serum inorganic phosphorus was decreased, indicating that it accelerated the bone tissue and bone transformation. Different doses of HSYA treatments reduced the level of ALP to a certain extent, proving its role in maintaining the balance between bone formation and bone resorption. The results of Giemsa staining showed that the cellular content in the bone marrow fluid was significantly higher after HSYA treatment than in the model group. In comparison, the range of fatty grains was significantly lower. The increase in bone marrow fat is essentially a marker of erythropoietic cytopenia (Griffith et al., 2012). Fat granules in bone marrow fluid indicate that estrogen deficiency causes mesenchymal stem cells to differentiate into adipocytes rather than hematopoietic cells or osteoblasts. In this study, HSYA and E2 can inhibit the differentiation of bone marrow mesenchymal stem cells into adipocytes and help to improve bone quality and bone biomechanical properties (Fonseca et al., 2021).

Deterioration in trabecular structure is associated with reduced bone strength and increased fracture incidence in humans. HE staining showed a significant increase in cartilaginous trabeculae and tibial thickness in the HSYA-treated group. The study found that in postmenopausal women, the trabecular plate and cortex began to thin, and then the microstructure of the trabecular and cortical bone deteriorated, leading to vertebral fractures. These microstructural defects would translate into lower bone stiffness at the femur and tibia. It is suggested that bone trabecular and microstructure abnormity may be essential in fracture susceptibility in postmenopausal women (Wang et al., 2016). Sirius red staining showed that HSYA was able to increase the amount of type I and III collagen, which is the main collagen in type I collagen bones, which is capable of conferring mechanical strength to the bone (Bailey et al., 1993). In the test results of the bone strength bearing threshold, the right leg bone of the model group reached a certain pressure threshold at the moment of fracture when tested by the INSTRON instrument. However, in the right leg bone of rats treated with HSYA, when the pressure reached a specific value and squeezed the bone tissue, no one-time fracture was observed. This could be due to there being a large amount of collagen in the bone tissue. The presence of large amounts of collagen increases the toughness of bone tissue. In addition, the reduction of trabecular bone and cortical thinning are important causes of bone strength reduction and fracture susceptibility.

Osteoblasts (OB) and osteoclasts (OC) are the two main cells that maintain bone mass in bone remodeling. Human bone growth and benign maintenance depend on the dynamic balance between bone resorption and bone formation. When the number of osteoclasts is greater than that of osteoblasts, osteoporosis or destruction of bone occurs. In this experiment, osteoblasts and osteoclasts were also stained and identified in the femoral tissue of each group of rats. As expected, osteoblasts were significantly reduced, osteoclasts were increased in the model group of rats, and HSYA effectively alleviated the imbalanced ratio of osteogenesis to Osteolysis. OB, derived from bone marrow mesenchymal stem cells, is an essential functional cell for bone formation, and its main function is to secrete bone matrix. Therefore, osteoclasts are essential to target cells in the treatment of osteoporosis (Fan et al., 2019).

Current evidence suggests that bone loss is caused by removing mineralized organic matrix by osteoclasts through interaction with other cell types, such as osteoblasts (Kylmaoja et al., 2016). Osteoclast bone resorption occurs in the central region of the contact area between the bone matrix and the cytoplasmic protrusions at the edge of the osteoclast folds. The extent of bone resorption depends on the secretion of acid and lysosomal enzymes. Carbonic anhydrase 2 (CA2) is a critical enzyme in osteoclast bone resorption and is the main producer of protons during osteoclast bone resorption CA2 is characteristically expressed in the early stages of osteoclast differentiation. Earlier studies have shown that CA2 can act as an intermediary to stimulate osteoclast differentiation (Laitala and Väänänen, 1993). Therefore, in conjunction with Autodock software, we predicted the binding site of HSYA to CA2. The alternative ligand site was a known inhibitor of CA2, aryl sulfonamide inhibitor, where multiple amino acid residue binding sites existed. Therefore CA2 was used as the initial target for our study. Surprisingly, subsequent qPCR in the model rats revealed that the gene expression level of CA 2 was indeed up-regulated, and we further verified this at the protein level. The results showed that estradiol alone decreased the protein expression levels of osteoclast differentiation markers RANKL, RANK (Park et al., 2017), and TRAF6/NFATc1/c-fos pathway (Kim et al., 2012; Chen et al., 2021), and decreased the protein expression levels of osteolysis related proteins CTSK (Lotinun et al., 2013), and MMP-9 (Zhu et al., 2020). In addition to reducing osteoclast differentiation markers and osteolysis-related proteins, HSYA treatment also reduced the expression level of CA2 protein to varying degrees. The expression of OPG protein was up-regulated. However, it has no significant effect on the protein expression level of CA2, and there is not much relevant literature on the impact of estradiol on CA2 (Zheng et al., 1995). We found only one study, also in osteoporosis, which reported that estradiol reduced the expression of CA2 in osteoporosis, which is different from our experimental findings. We analyzed the experiment by Zheng et al. in which estradiol injection for 18 h reduced the gene expression of CA2 in osteoclasts of osteoporotic rats. This inhibition of osteoclastic bone resorption enzymes was most effective in the first 1–2 weeks, with the degree of inhibition decreasing 8 weeks after ovariectomy. In contrast, our experiment was performed by gavage administration of estradiol 1 week after ovariectomy, and the administration lasted up to 3 months. The factors that contributed to the discrepancy are unclear. 

From this analysis, it is clear that HSYA does have a regulatory effect on CA2 and that the inhibitory effect of HSYA on CA2 has a long-term impact compared to estradiol. In addition, we conducted induction studies at the osteoclast level, and the RAW 264.7 mouse cell line was proven to be an essential tool for *in vitro* studies of osteoclast formation and function (Collin-Osdoby and Osdoby, 2012). Therefore, we used RAW264.7 cells for osteoclast induction, and western blot detection proved that HSYA could inhibit the differentiation of RAW264.7 cells into osteoclasts. The therapeutic effect of HSYA interfered with mouse CA2 recombinant protein. These results suggest that CA2 can effectively reverse the inhibition of HSYA on osteoclast differentiation. Therefore, we believe that HSYA can inhibit the differentiation of osteoclasts by inhibiting the expression of CA2 from achieving the therapeutic effect of osteoporosis.

Osteoporosis is closely associated with abnormally active osteoclast differentiation, proliferation, and bone resorption. Among these, CA2 plays an important role. In this study, an osteoporosis model was first constructed by depleted female rats. The protective effect of HSYA on osteoporotic rat’s bone mass was determined by late bone histopathological tests and serum index. Combined with literature and molecular docking, CA2 was an entry point to explore its regulatory role in osteoclast differentiation and function. The ability of HSYA to inhibit the function of osteoclasts through the inhibition of CA2 expression was verified by western blotting and other experiments. Combined with cell-level experiments, we conducted studies on the regulation of HSYA on CA2 and osteoclast differentiation mechanism and proved that HSYA could inhibit osteoclast differentiation by inhibiting CA2 protein. Based on the safety of HSYA for consumption, HSYA can be used as a preventive health drug in the treatment of osteoporosis.

## Data Availability

The datasets used and/or analyzed during the current study are available from the corresponding author on reasonable request.

## Supplementary Material

Supplementary Figure 1 Results of Gipsa staining of vaginal secretions in rats. **(A)** Rats in the model group **(B)** Rats in the depot group.

Supplementary Figure 2 Morphology of rat ovaries after intervention.

Supplementary Figure 3 Femoral strength test method in rats.
