# Supplementary figures and images for: Hydroxy-Safflower Yellow A Alleviates Osteoporosis in Ovariectomized Rat Model by Inhibiting Carbonic Anhydrase 2 Activity
Source: Front Pharmacol. 2021 Nov 5;12:734539. doi: 10.3389/fphar.2021.734539 (PMC8602693; doi:10.3389/fphar.2021.734539)

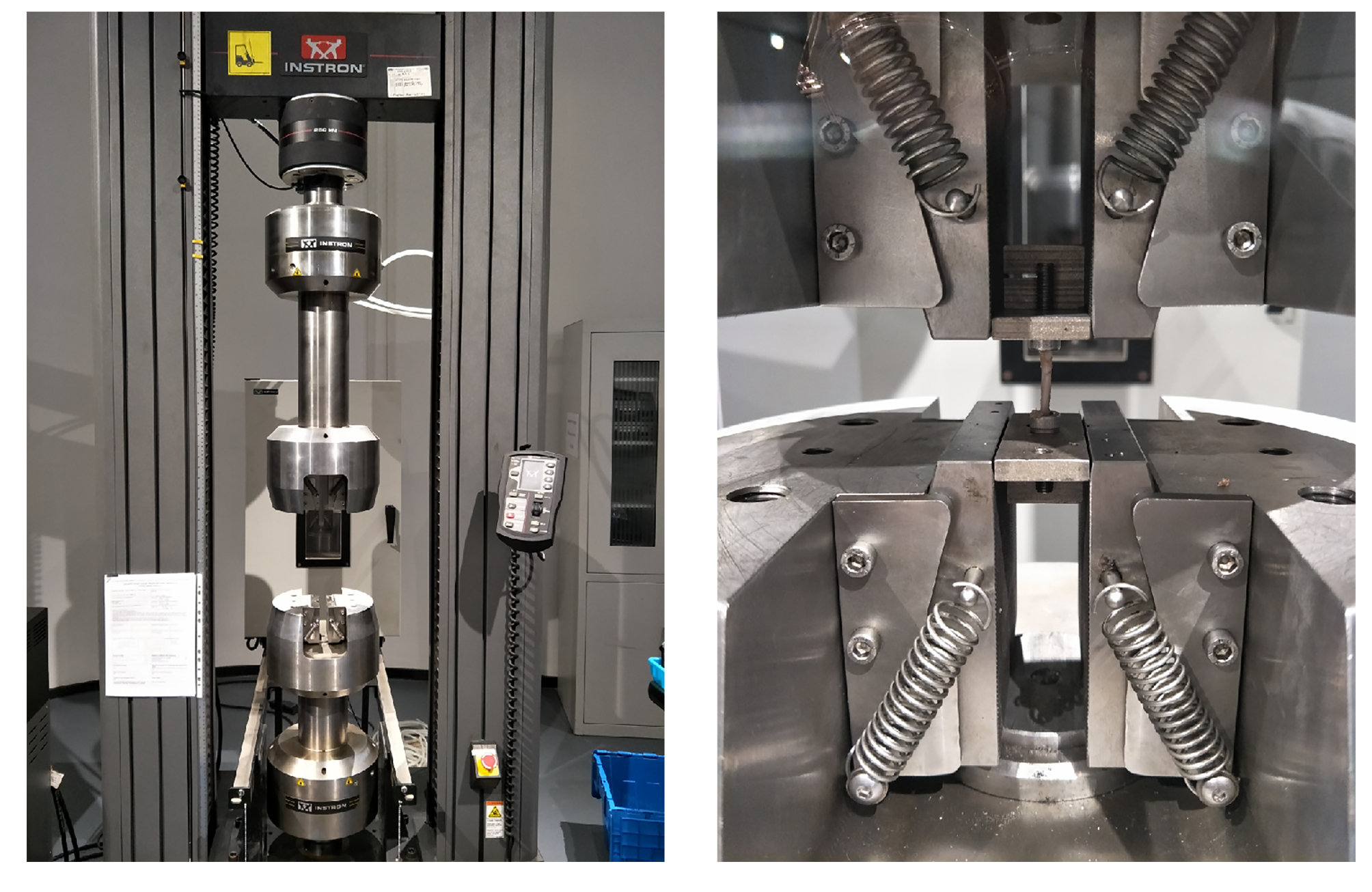

Supplement: Supplementary file 1 [file Image3.TIF]

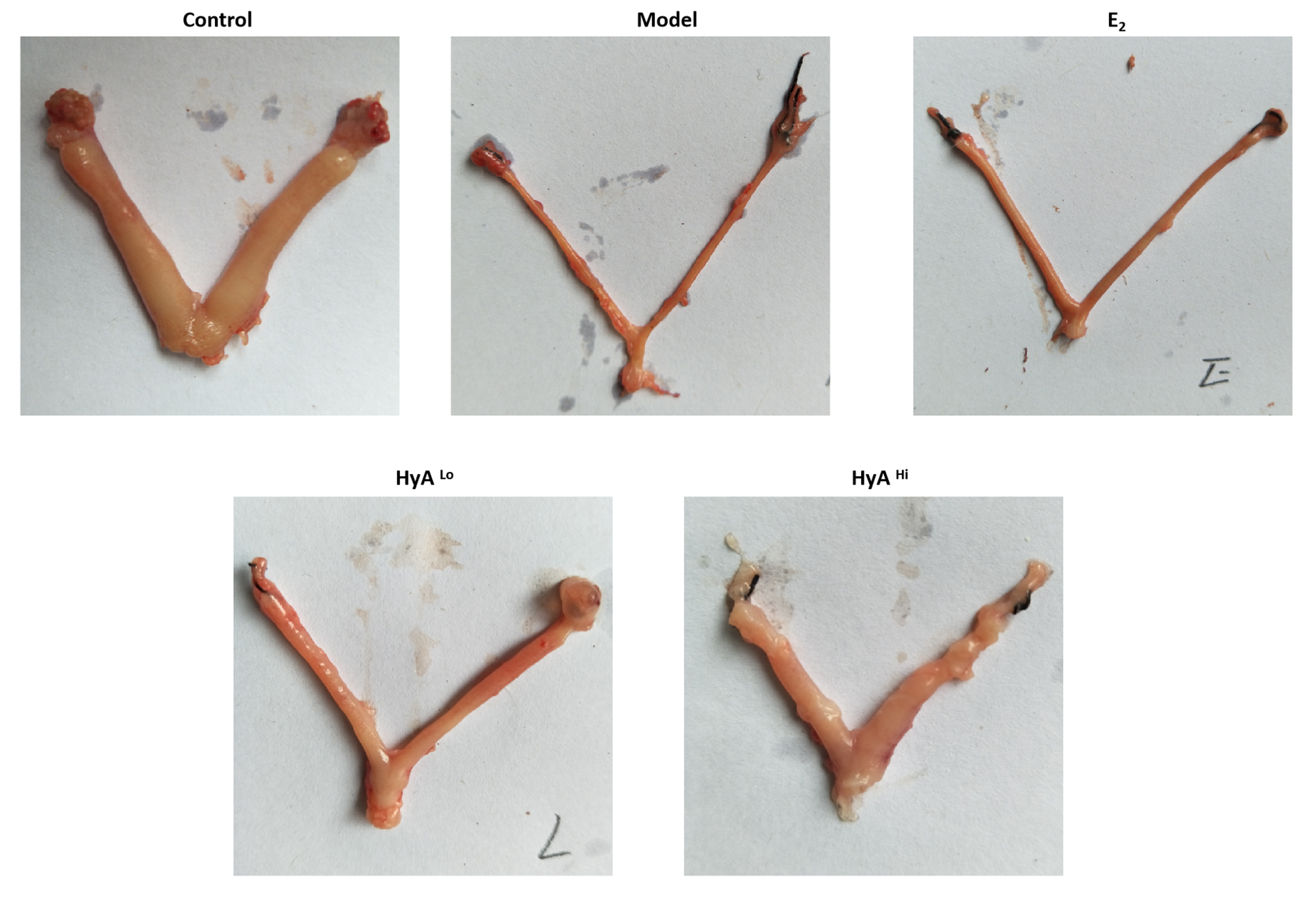

Supplement: Supplementary file 2 [file Image2.TIF]

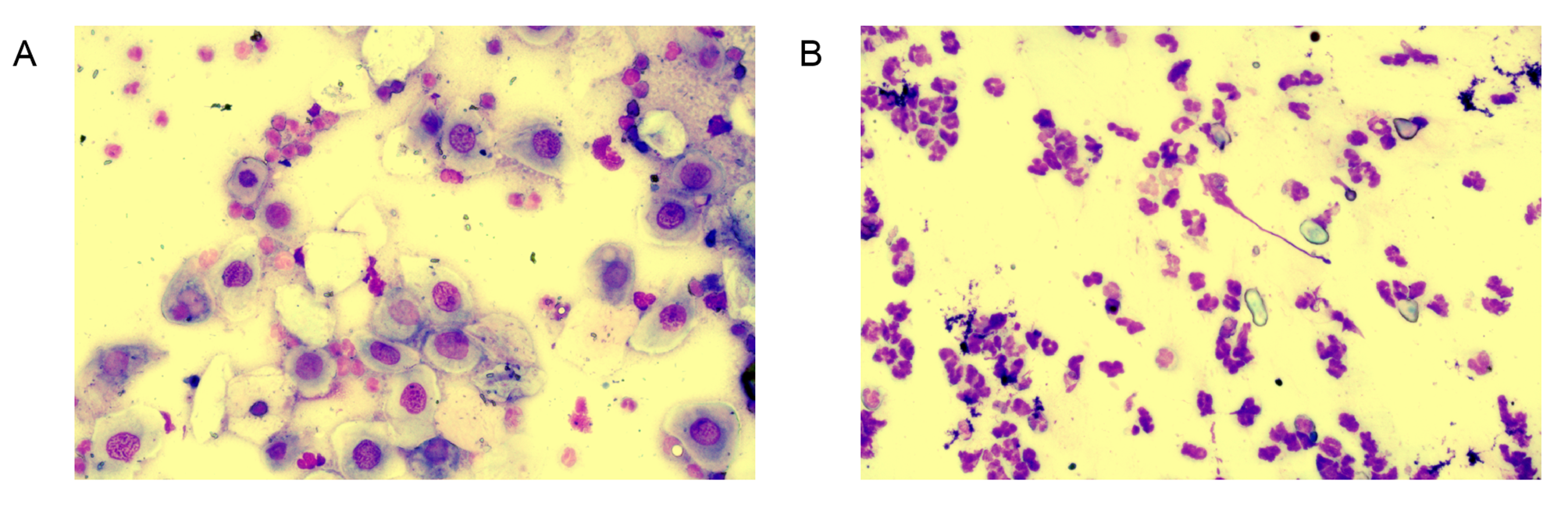

Supplement: Supplementary file 3 [file Image1.TIF]
